# Characterization of the Maize GASA Gene Family and Their Responses to Low-Phosphorus Stress

**DOI:** 10.3390/plants15020309

**Published:** 2026-01-20

**Authors:** Chaoye Dao, Feiyan Li, Shuang Li, Zengqiang Meng, Litao Yi, Qiuyue Yang, Weiwei Huang, Xiupeng Mei, Jiuguang Wang, Chaofeng Li

**Affiliations:** 1 Maize Research Institute, Southwest University, Chongqing 400715, China; cairosnow@outlook.com (C.D.); lifeiyan200103@163.com (F.L.); ls18855077417@126.com (S.L.); 17782177173@163.com (Z.M.); 13068332389@163.com (L.Y.); yqyypkz8299@163.com (Q.Y.); mei2021@swu.edu.cn (X.M.); 2 College of Agronomy and Biotechnology, Southwest University, Chongqing 400715, China; 3 Co-Innovation Center for Sustainable Forestry in Southern China, Bamboo Research Institute, College of Forestry and Grassland, Nanjing Forestry University, Nanjing 210037, China; wh@njfu.edu.cn; 4 Department of Geosciences and Natural Resource Management, The University of Copenhagen, Rolighedsvej 23, DK-1958 Frederiksberg, Denmark

**Keywords:** *Zea mays*, GASA, low phosphorus, stress response

## Abstract

Gibberellic Acid-Stimulated Arabidopsis (GASA) proteins are cysteine-rich peptides broadly conserved in plants and implicated in growth regulation, stress adaptation, and hormone signaling. Here, we identified 13 *GASA* genes in the maize genome, distributed across six chromosomes. Comprehensive analyses of their physicochemical properties, subcellular localization, and structural features classified them into three conserved clades. Promoter cis-element analysis suggested roles in developmental regulation, phytohormone responses, abiotic stress adaptation, and light signaling. Comparative synteny revealed close evolutionary relationships between maize and sugarcane *GASA* genes, implying divergence from a common ancestor. Transcriptomic and qRT-PCR analyses demonstrated that maize GASA genes exhibit tissue-specific and stress-responsive expression under low-phosphorus condition, with *ZmGASA06* showing pronounced responsiveness to low-phosphorus stress. This study provides novel insights into the evolutionary dynamics and potential functional roles of the GASA family in maize, laying a foundation for further functional and molecular breeding studies.

## 1. Introduction

The Gibberellic Acid-Stimulated *Arabidopsis* (GASA) gene family constitutes a plant-specific group encoding small cysteine-rich proteins, broadly conserved across gymnosperms, angiosperms, and ferns [1]. Most GASA proteins are secreted to the cell wall and share a conserved structural architecture, including an N-terminal signal peptide, a hydrophilic central region, and a highly conserved cysteine-rich C-terminal domain [2]. The defining feature of the GASA domain is a 12-cysteine motif (XnCX3CX2RCX8CX3CX2CCX2CXCVPXGX2GNX3CPCYX10KCP) [3], which has remained evolutionarily stable. Mutational analyses have shown that disruption of critical cysteine residues abolishes GASA functionality, highlighting the indispensable role of this motif in plant growth and stress adaptation.

Since their first identification in tomato (*Solanum lycopersicum*) [4], GASA genes have been reported in diverse species such as potato (*S. tuberosum*) [5], poplar (*Populus trichocarpa*) [6], and Arabidopsis thaliana [7]. The Arabidopsis genome encodes 14 GASA members (AtGASA1–AtGASA14), several of which (e.g., AtGASA4, AtGASA5, AtGASA6, AtGASA10, AtGASA14) have been functionally characterized. These studies indicate that GASA proteins act at the intersection of hormone signaling, protein–protein interaction, and redox regulation [8,9,10], thereby integrating developmental and environmental cues. Functional diversification within the family is evident. For example, AtGASA4 enhances thermotolerance by restricting ROS accumulation; AtGASA6 promotes early flowering but is repressed by hormones, such as ABA, JA, and SA [7]; AtGASA5 mediates heat sensitivity in a GA-dependent manner; and AtGASA14, with its unique proline-rich N-terminal extension, promotes ABA accumulation when overexpressed [11].

Beyond *Arabidopsis*, *GASA* genes have been implicated in growth and morphogenesis, hormone crosstalk, cell proliferation, seed development, and abiotic stress responses in multiple plant species, such as *Hordeum vulgare* L., tomato, *Populus*, *Nicotiana tabacum*, *Medicago truncatula*, *Canavalia rosea*, cucumber, and sunflower [4,6,8,10,11,12,13,14,15,16,17]. As downstream targets of DELLA proteins, *GASA* genes are regulated by gibberellins (GAs), which promote plant growth by inducing DELLA protein degradation and are widely involved in seed germination, organ elongation, trichome development, phase transition, and reproductive growth [18]. Elucidating the structure and function of *GASA* genes is thus critical for advancing our understanding of GA signaling and its interaction with other hormonal pathways. In addition to GAs, *GASA* genes also respond to auxin, cytokinins, brassinosteroids, ethylene, ABA [19], JA, and SA [20], underscoring their central role in hormone regulatory networks. For instance, in *Prunus mume* (Rosaceae), *PmGASA1* and *PmGASA11* are responsive to exogenous IAA, GA, and ABA [21], while in *Brassica rapa* (Brassicaceae), *BrGASA13* and *BrGASA5* harbor drought-inducible cis-elements (e.g., MBS and DRE) in their promoters, contributing to drought tolerance [22]. In addition, *GASA* genes participate in GA-mediated flowering pathways, influencing flowering time and stability [23], and regulate cell division and elongation to control the morphogenesis and growth of roots, stems, leaves, and fruits [24].

Furthermore, GASA proteins exhibit antimicrobial peptide (AMP)-like activity, contributing to defense against bacterial and fungal pathogens [11]. With growing recognition of cysteine-rich peptides as pivotal regulators in plant biology, GASA proteins are now considered key coordinators of developmental programs and adaptive responses to both biotic and abiotic stresses, and have been identified in both monocot and dicot lineages.

Maize (*Zea mays* L.), a major cereal crop with high yield potential and wide adaptability, also serves as a model species for genetic and functional research. Despite advances in identifying hormone- and stress-responsive genes in maize, the roles of GASA family members in maize growth, development, and abiotic stress responses remain largely unknown.

Phosphorus is a non-renewable resource, and reducing its input is essential for sustainable agriculture [25]. Therefore, applying low-phosphorus treatment enables the investigation of maize adaptive and regulatory responses to contrasting nutrient availability. In this study, low-phosphorus and treatment was used to identify key *GASA* genes. This study presents the first comprehensive characterization of the maize *GASA* gene family, providing insights into their potential functions and evolutionary significance across plant species.

## 2. Results

### 2.1. Genome-Wide Identification of the Maize GASA Gene Family

Through BLAST (Version 2.17.0), SMART(Version 10), and InterPro (Version 107.0) analyses, a total of 13 *ZmGASA* genes were identified in the maize genome and sequentially designated *ZmGASA01* to *ZmGASA13* according to their chromosomal positions. These genes represent the full complement of the maize GASA gene family.

Predictions of transmembrane domains and their topological structures were performed using bioinformatics platforms. In addition, the physicochemical properties of ZmGASA proteins were analyzed, including amino acid composition, protein length, molecular weight, and isoelectric point (pI). The results are summarized in Table 1. Most family members contain 95–300 amino acids, with molecular weights ranging from 9733.7 to 22,314.63 Da (average: 12,447.25 Da). Among them, ZmGASA09 encodes the longest protein with the highest molecular weight (22,314.63 Da), whereas ZmGASA04 encodes the shortest protein with the lowest molecular weight (9733.7 Da). The coding sequence (CDS) lengths vary between 282 and 600 bp. Subcellular localization prediction indicated that ZmGASA proteins are predominantly localized to the cytoplasm (7 genes) and chloroplasts (6 genes). With the exception of ZmGASA01 and ZmGASA07, which exhibit relatively low pI values, most ZmGASA proteins have isoelectric points between 8 and 10 (average: 8.58), suggesting a predominantly basic nature. Notably, the pronounced differences in amino acid length and molecular weight between ZmGASA04 and ZmGASA09 imply potential structural and functional divergence between these two members.

### 2.2. Secondary Structure and Tertiary Structure Modeling of Maize GASA Proteins

**Figure 1:**
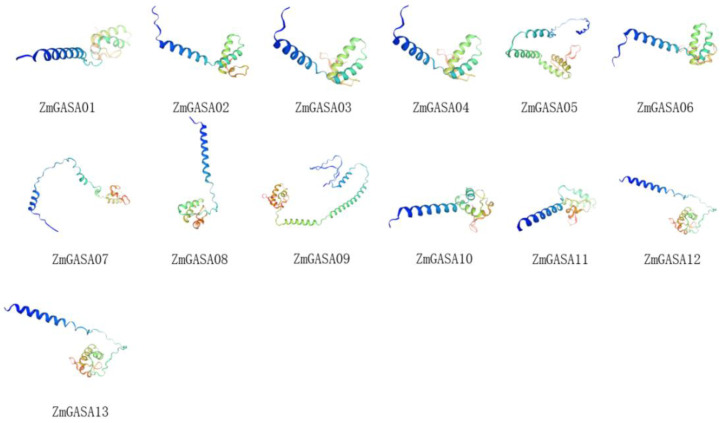
Predicted the tertiary structures of maize ZmGASA proteins. Blue colour represents the N-terminus of the amino acid sequence, and red colour represents the C-terminus of the amino acid sequence. Then, the blue part is an alpha-helix, and the green (light yellow) part is a beta-sheet.

Prediction analysis revealed four secondary structure elements of maize GASA proteins, including α-helices, extended strands, β-turns, and random coils, with their respective numbers and proportions summarized in Table 2. In addition, tertiary structure models of the 13 ZmGASA proteins were generated (Figure 1). The results indicated that α-helices and random coils accounted for the largest proportions of secondary structures. Specifically, the number of α-helices ranged from 19 to 62, with an average proportion of 32.56%, whereas random coils ranged from 39 to 96, with an average proportion of 51.35%. By contrast, extended strands and β-turns were less abundant, each comprising less than 20% of the total. These findings suggest that α-helices and random coils are the predominant structural components of ZmGASA proteins.

**Figure 2:**
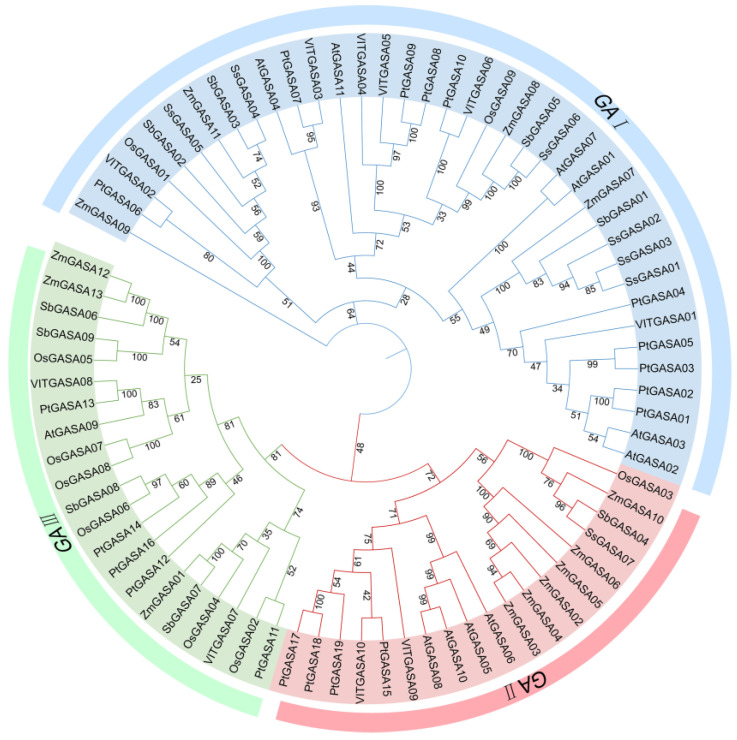
Phylogenetic analysis of GASA family proteins from maize and six other plant species. The outer circle of the phylogenetic tree denotes the subfamily classification of *GASA* genes, the middle circle shows the corresponding gene names, and the inner circle indicates the specific clades to which each gene belongs. Numbers at the nodes represent bootstrap support values based on 1000 replicates. The species included are *Zea mays* (Zm), *Arabidopsis thaliana* (Ath), *Oryza sativa* (Os), *Sorghum bicolor* (Sb), *Saccharum officinarum* (So), *Vitis vinifera* (Vv), and *Populus trichocarpa* (Ptr).

**Figure 3:**
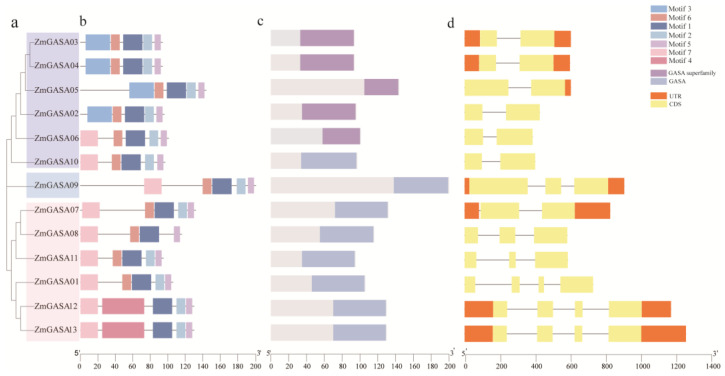
Conserved motifs and gene structures of maize GASA family members. (**a**) Phylogenetic tree of GASA proteins; (**b**) conserved amino acid motifs identified in GASA proteins; (**c**) GASA domain architecture; (**d**) gene structure analysis showing coding sequences (CDSs), introns, and untranslated regions (UTRs).

Tertiary structure modeling further demonstrated that although the overall conformations of individual proteins differ, they are generally consistent with secondary structure predictions and all contain a conserved helix-turn-helix (HTH) motif. Importantly, members clustered within closer evolutionary relationships (Figure 2 and Figure 3a) exhibited highly similar tertiary structures. For instance, ZmGASA02, ZmGASA03, ZmGASA04, ZmGASA05, and ZmGASA10 shared similar structural features (Figure 1, Figure 2 and Figure 3a), while ZmGASA01, ZmGASA07, ZmGASA08, ZmGASA11, ZmGASA12, and ZmGASA13 also displayed comparable conformations within another branch (Figure 1, Figure 2 and Figure 3a). These results imply that ZmGASA proteins within the same phylogenetic clade may possess analogous structural features and potentially similar biological functions.

### 2.3. Phylogenetic Analysis of GASA Family Members Across Different Species

Phylogenetic analysis revealed that *GASA* genes from seven species can be classified into three subfamilies (GAI, GAII, and GAIII). Members from all seven species were distributed across the three subfamilies. In maize, the GAI subfamily comprises four members (*ZmGASA09*, *ZmGASA11*, *ZmGASA07*, and *ZmGASA08*), the GAII subfamily includes six members (*ZmGASA02*, *ZmGASA03*, *ZmGASA04*, *ZmGASA05*, *ZmGASA06*, and *ZmGASA10*), and the GAIII subfamily consists of three members (*ZmGASA01*, *ZmGASA12*, and *ZmGASA13*). These findings indicate that the functions of *GASA* genes are relatively conserved within subfamilies across different species.

Moreover, the phylogenetic distribution of maize GASA family members was found to be highly similar to that of sorghum and sugarcane, suggesting a closer evolutionary relationship among these species and implying a more recent common ancestor.

### 2.4. Phylogenetic Tree, Conserved Motifs, Conserved Domains, and Gene Structure Analyses of the Maize GASA Gene Family

Using MEGA 11 software, a phylogenetic tree of the maize GASA gene family was constructed based on the amino acid sequences encoded by the 13 *ZmGASA* genes. The results showed that the ZmGASA family members could be classified into three major groups, containing three pairs of paralogous genes, *ZmGASA03/ZmGASA04*, *ZmGASA07/ZmGASA08*, and *ZmGASA12/ZmGASA13* (Figure 3a), which is largely consistent with the grouping observed in Figure 2.

Conserved motif prediction identified seven motifs (Figure 3b). Among them, Motif 1, Motif 2, and Motif 5 were present in all family members. Motif 3 was uniquely detected in ZmGASA02, ZmGASA03, ZmGASA04, and ZmGASA05, whereas Motif 6 was identified in 11 members, excluding ZmGASA12 and ZmGASA13. Motif 7 was observed in ZmGASA01, ZmGASA06, ZmGASA07, ZmGASA08, ZmGASA09, ZmGASA10, ZmGASA11, ZmGASA12, and ZmGASA13. Notably, Motif 4 was specifically present in ZmGASA12 and ZmGASA13, suggesting that these two genes may play specialized roles within the family (Figure 3b).

To further investigate their potential functions, gene structure analysis was performed (Figure 3d). The results revealed three structural patterns: *ZmGASA02*, *ZmGASA03*, *ZmGASA04*, *ZmGASA05*, *ZmGASA06*, *ZmGASA07*, and *ZmGASA10* share a gene structure with two exons and one intron; *ZmGASA08*, *ZmGASA09*, and *ZmGASA11* have three exons and two introns; *ZmGASA01*, *ZmGASA12*, and *ZmGASA13* contain four exons and three introns.

The relatively simple exon–intron organization of *ZmGASA02*, *ZmGASA03*, *ZmGASA04*, *ZmGASA05*, *ZmGASA06*, *ZmGASA07*, and *ZmGASA10* suggests that these genes may have diverged later during evolution and could be functionally more specialized. In addition, pairs such as *ZmGASA03/ZmGASA04* and *ZmGASA12/ZmGASA13* exhibited highly similar gene structures and strong homology in the phylogenetic tree, indicating that closely related genes often retain conserved structural features during evolution.

Furthermore, conserved domain analysis (Figure 3c) revealed that all family members contain the characteristic GASA domain (XnCX3CX2RCX8CX3CX2CCX2CXCVPXGX2GNX3CPCYX10KCP). Among them, ZmGASA03, ZmGASA04, ZmGASA05, ZmGASA02, ZmGASA06, and ZmGASA10 harbor the GASA superfamily domain, while ZmGASA10, ZmGASA09, ZmGASA07, ZmGASA08, ZmGASA11, ZmGASA01, ZmGASA12, and ZmGASA13 contain the GASA domain. Comparative analysis further indicated that Motif 1 (PGBIHDCPCYRNMLT), Motif 2 (MAKPPLQTAAIILLVLLAAASCLHTVDAAALGFCWGKCSVRCARATARQA), Motif 5 (PKKRPKCP), and Motif 6 (FLFLAAVAASAAEMIAGSGIGDGEGEELD) constitute the core components of the GASA superfamily domain.

### 2.5. Cis-Acting Element Analysis of Maize GASA Promoters

**Figure 4:**
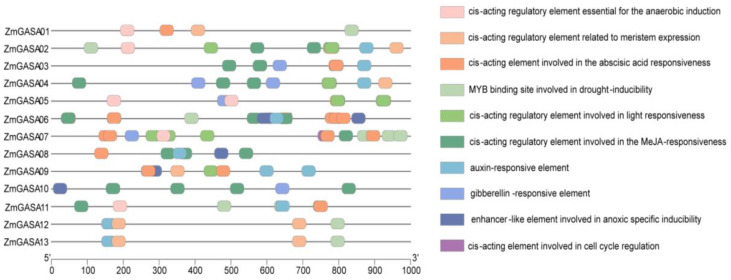
Cis-acting element analysis of the promoter regions of *ZmGASA* genes in maize. The right panel displays the types of cis-regulatory elements identified in the promoter regions, while the left panel illustrates their positional distribution along the *ZmGASA* gene promoters.

To further investigate the functions and regulatory mechanisms of *ZmGASA* genes, the promoter regions (2000 bp upstream of the transcription start site) of 13 *ZmGASA* genes were analyzed. The results revealed that the promoter regions of all *ZmGASA* genes contain multiple cis-acting elements associated with plant growth and development, hormone responsiveness, stress responses, and light signaling (Figure 4), suggesting that *ZmGASA* genes play important roles in plant growth, development, and environmental adaptation.

In addition to the core promoter elements TATA-box and CAAT-box, the promoters of *ZmGASA* genes harbor numerous functional elements, which can be classified into hormone-responsive elements, stress-related elements, light-responsive elements, and growth-related elements. In total, 144 cis-acting elements were identified across the 13 *ZmGASA* promoters, including 85 hormone-responsive elements: 30 abscisic acid (ABA)-responsive elements, 39 methyl jasmonate (MeJA)-responsive elements, 10 auxin-responsive elements, and 6 gibberellin (GA)-responsive elements. Among them, ABA-responsive elements were enriched in *ZmGASA06* and *ZmGASA05*, while MeJA-responsive elements were particularly abundant in *ZmGASA06*. Most family members contained auxin- and MeJA-responsive elements.

A total of 19 stress-related elements were identified, including 5 anaerobic induction elements, 9 drought-inducible elements, and 5 anoxic induction enhancers. *ZmGASA07* possessed the highest number of drought-inducible elements (3), whereas *ZmGASA06* contained a greater number of anoxic induction enhancers. Additionally, 31 light-responsive elements were identified, with relatively higher frequencies in *ZmGASA02* and *ZmGASA07*. With respect to growth-related elements, 9 were detected, including 8 meristem-specific expression elements predominantly found in *ZmGASA12* and *ZmGASA13*. Notably, these two genes also harbored a greater number of auxin-responsive elements, suggesting that *ZmGASA12* and *ZmGASA13* may be closely involved in the regulation of plant growth and development. Interestingly, only one cell cycle regulatory element was detected, exclusively in the promoter of *ZmGASA07*, indicating that this gene may play a specialized role within the family.

Taken together, these results demonstrate that auxin-responsive elements and MeJA-responsive elements are widely distributed across the ZmGASA family, while cell cycle regulatory elements, anaerobic response elements, and anoxic response elements are concentrated in a few specific members. This result suggests that the regulatory mechanisms of *ZmGASA* genes share both common features and member-specific specializations, which underlie their fundamental and specialized biological functions.

### 2.6. Inter-Species Synteny Analysis of the Maize GASA Gene Family

**Figure 5:**
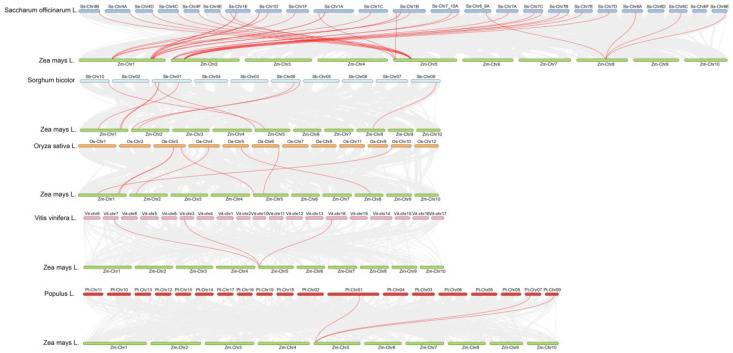
Collinearity analysis of GASA gene family among between maize and sugarcane, sorghum, grape, rice and poplar. Gray lines indicate syntenic gene pairs between maize and *Saccharum officinarum,* (*Oryza sativa*, *Sorghum bicolor*, *Vitis vinifera*, or *Populus trichocarpa*), while bold red lines represent co-syntenic gene pairs of the GASA gene family between maize and other plant species.

To further elucidate the evolutionary relationships of *ZmGASA* genes across species, a synteny analysis was performed between maize and rice, sorghum, sugarcane, grape, and poplar (Figure 5). The results revealed that 30 homologous gene pairs were identified between 4 maize chromosomes and 22 sugarcane chromosomes, 8 homologous gene pairs between 4 maize chromosomes and 4 sorghum chromosomes, 3 homologous gene pairs between 1 maize chromosome and 3 grape chromosomes, 7 homologous gene pairs between 4 maize chromosomes and 5 rice chromosomes, and 3 homologous gene pairs between 1 maize chromosome and 3 poplar chromosomes.

Notably, the syntenic relationships were most abundant between maize and sugarcane as well as between maize and sorghum, indicating that the maize *GASA* genes are more closely related to those in sugarcane and sorghum. These findings suggest that they likely originated from a common ancestral lineage and may have retained conserved functional roles during evolution.

### 2.7. Expression Profiling of the Maize GASA Gene Family Under Stress Conditions

Phosphorus is a non-renewable resource, and reducing phosphorus input while improving phosphorus-use efficiency will be an inevitable strategy in future maize production systems. Therefore, in this study, three maize inbred lines (082, Ye107, and B73) were subjected to low-phosphorus (LP) stress at the three-leaf stage and identified the key *GASA* genes. After 14 days of treatment, we determined the growth-related physiological parameters of maize seedlings, including plant height, fresh weight, and dry weight. Although distinct growth differences were observed among the three inbred lines, these variations were statistically non-significant and thus not presented in this manuscript. But some *GASA* genes exhibit substantial differences at the transcriptional levels. The results revealed that most ZmGASA family members exhibited differential transcriptional regulation under both conditions.

**Figure 6:**
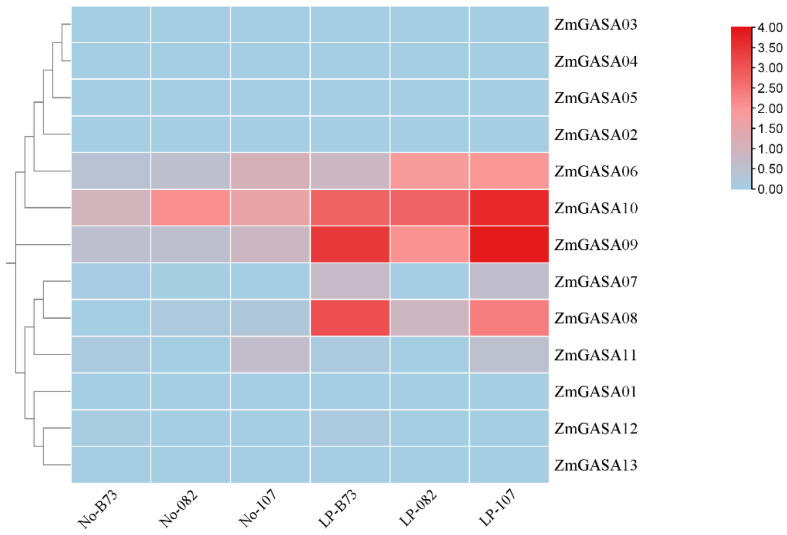
Heatmap showing the expression profiles of maize GASA family genes under low-phosphorus treatment. No indicates maize seedling samples grown under normal nutrient conditions, LP represents seedlings grown under low-phosphorus conditions. Blue, gray, and red boxes indicate low, moderate, and high expression levels, respectively.

Under LP stress: In inbred line 082, *ZmGASA06*, *ZmGASA08*, *ZmGASA09*, and *ZmGASA10* were significantly upregulated, with *ZmGASA08* and *ZmGASA09* reaching 5.82-fold and 3.77-fold higher expression than controls, respectively; In line Ye107, *ZmGASA06*, *ZmGASA07*, *ZmGASA08*, *ZmGASA09*, and *ZmGASA10* were upregulated, with *ZmGASA08* showing the strongest induction (9.49-fold), whereas *ZmGASA11* was downregulated; In line B73, *ZmGASA06*, *ZmGASA07*, *ZmGASA08*, *ZmGASA09*, *ZmGASA10*, and *ZmGASA12* were upregulated, with *ZmGASA07* exhibiting a dramatic induction (16.60-fold compared with control) (Figure 6). Taken together, *ZmGASA08*, *ZmGASA09*, and *ZmGASA10* were consistently induced by LP stress across all three inbred lines, suggesting their critical involvement in maize phosphate-deficiency adaptation.

**Figure 7:**
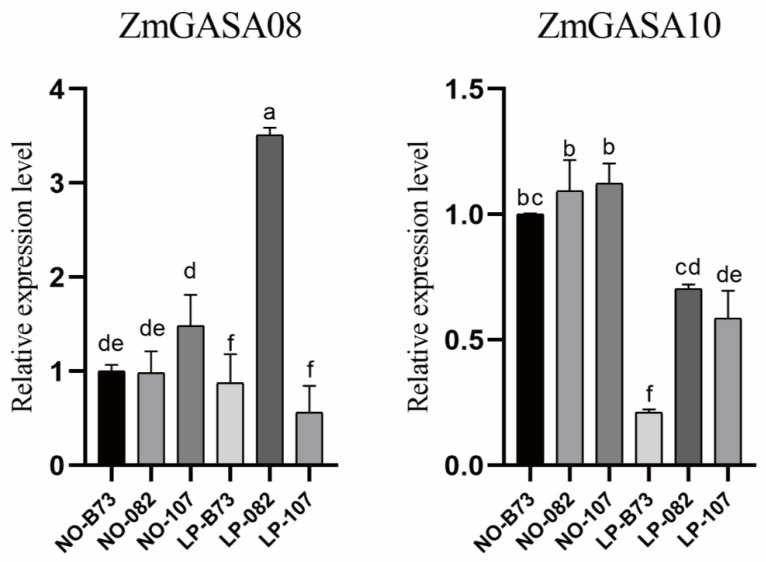
Relative expression levels of *ZmGASA08* and *ZmGASA10* genes with qRT-PCR analysis. No indicates maize seedling samples grown under normal nutrient conditions, LP represents seedlings grown under low-phosphorus conditions. Different letters above the bars indicate statistically significant differences among treatments (*p* < 0.05), as determined by one-way ANOVA followed by the LSD and Waller–Duncan post hoc tests.

QRT-PCR assays of *ZmGASA08* and *ZmGASA10* confirmed that they were strongly induced in lines 082 and Ye107under LP stress, while in line B73, their expression was moderately elevated (Figure 7).

Collectively, these results provide important insights into the functional diversification and regulatory mechanisms of *ZmGASA* genes in nutrient stress adaptation.

## 3. Discussion

GASA gene family is widely distributed across plant taxa and encode cysteine-rich peptide (CRP) proteins. These proteins are typically characterized by an N-terminal signal peptide and a conserved C-terminal GASA domain [1]. Members of the GASA gene family have been demonstrated to play essential roles in plant growth and development, responses to biotic and abiotic stresses, and phytohormone signaling pathways [7].

To date, the biological functions and bioinformatic characterization of *GASA* genes have been extensively studied in horticultural crops and ornamentals, such as tomato [7], Chinese cabbage [7], chrysanthemum [7], rose [7], and crape myrtle [7], whereas reports in cereal crops remain limited. In this study, based on the 14 members of the *Arabidopsis* GASA gene family [7], 13 *GASA* genes were identified in maize through genome-wide screening, followed by structural and expression analyses. Notably, certain *Arabidopsis GASA* homologs were absent in maize, suggesting potential gene loss events in maize or gene acquisition in *Arabidopsis* during evolution. The identified maize *GASA* genes were unevenly distributed across six chromosomes, with the majority located on chromosome 1. This distribution pattern differs from *Arabidopsis* but is consistent with other species such as potato [6], pineapple [24], and poplar [6].

Subcellular localization predictions indicated that most ZmGASA proteins localize to the cytoplasm, while a subset is targeted to chloroplasts. This finding contrasts with poplar GASA proteins, which are localized to the cell wall, nucleus, plasma membrane, and Golgi apparatus [6], implying potential functional divergence. Protein secondary and tertiary structure analyses revealed that ZmGASA proteins share common structural features, including α-helices, extended strands, β-turns, and random coils, with α-helices and random coils being predominant. These findings are consistent with structural predictions for poplar and pineapple GASAs [6,24].

Phylogenetic analysis of seven plant species showed that GASA members clustered into three major groups, with clade GAI containing the largest number of genes. Gene structure analysis revealed that certain members (e.g., *ZmGASA02*, *ZmGASA06*, *ZmGASA10*, *ZmGASA08*, *ZmGASA11*, and *ZmGASA01*) possess relatively simple architectures composed of only two or three coding sequences (CDSs), suggesting their relatively recent origin and functional specialization. Pairs of genes such as *ZmGASA03*/*04* and *ZmGASA12/13* exhibited highly similar structures, consistent with their strong homology in phylogenetic trees. Conserved motif analysis further revealed that Motifs 1, 2, and 5 were universally present across all members, likely contributing to core GASA functions, while other motifs (e.g., Motif 3 and Motif 4) were restricted to specific subgroups, implying functional diversification.

Cis-acting regulatory element analysis identified 144 elements across 13 *ZmGASA* promoters, including those associated with growth and development, phytohormone signaling, stress responses, and light responsiveness. Among these, abscisic acid (ABA)-responsive and methyl jasmonate (MeJA)-responsive elements were the most abundant, suggesting a central role in ABA and MeJA signaling. Interestingly, *ZmGASA04* harbored cis-elements responsive to ABA, MeJA, auxin, and gibberellins, indicating a possible hub role in cross-talk between multiple hormone pathways—since the presence of a cis-element does not prove but only shows the possibility. Previous studies in *Arabidopsis* have shown that *AtGASA01* and *AtGASA04* participate in hormone signaling [7]. Although *GASA* genes are generally GA-inducible [7], only *ZmGASA03*, *ZmGASA04*, *ZmGASA07*, and *ZmGASA10* contained GA-responsive elements, suggesting that GA regulation in maize might be indirect. Notably, *ZmGASA07* contained a unique cell cycle regulatory element, implying a specialized role. Moreover, in agreement with previous studies linking *GASAs* to reactive oxygen species (ROS) dynamics [11], maize *GASAs* may contribute to ROS accumulation, promoting cell expansion and enhancing abiotic stress tolerance.

Comparative synteny analysis revealed strong collinearity between maize and sugarcane, with 30 homologous gene pairs identified across four maize and 22 sugarcane chromosomes. This number exceeded syntenic gene pairs between maize and sorghum, grape, rice, or poplar, indicating a closer evolutionary relationship between maize and sugarcane GASAs. This finding suggests that maize and sugarcane GASAs likely evolved from a common ancestor and may retain conserved biological functions.

Maize requires an adequate and balanced supply of mineral nutrients—particularly nitrogen (N), phosphorus (P), and potassium (K)—to sustain optimal growth, development, and high grain yield. Among these macronutrients, phosphorus plays especially critical roles in maize productivity [25,26]. Phosphorus is essential for energy transfer, signal transduction, and membrane synthesis, and it is particularly important for root development, flowering, and grain formation [27,28,29]. Insufficient availability of phosphorus can severely constrain vegetative growth, delay developmental processes, and ultimately reduce grain yield and quality in maize production systems [30]. In this study, to identify the key GASA genes improving maize growth, the expression profiling under nutrient stress showed differential regulation of *ZmGASA* genes. *ZmGASA06* was consistently upregulated in three maize inbred lines under low-phosphorus condition, highlighting its importance in stress adaptation of the particular stresses. Additionally, *ZmGASA08*, *ZmGASA09*, and *ZmGASA10* were induced by low-phosphorus stress across all three inbred lines.

In summary, this study identified 13 maize *GASA* genes, classified into three phylogenetic groups, with most members localized on chromosomes 1, 2, 5, and 8. No tandem or segmental duplication events were detected. Synteny analysis revealed closer evolutionary relationships between maize and sugarcane GASA families. Cis-regulatory element profiling suggested roles in hormone and stress signaling, while expression analyses highlighted specific members responsive to nutrient stress. Collectively, these findings provide valuable insights into the evolutionary dynamics and functional roles of ZmGASAs and offer genetic resources for future studies on maize adaptation to abiotic stresses.

## 4. Materials and Methods

### 4.1. Genome-Wide Identification of GASA Gene Family Members in Maize

The genomic and coding sequences (CDS) of maize GASA family members (Zm-B73-REFERENCE-NAM-5.0.55) and *Arabidopsis* thaliana GASA family members (TAIR10) were retrieved from the Phytozome 13 database (https://phytozome-next.jgi.doe.gov, accessed on 14 December 2024). Using the reported *Arabidopsis* GASA proteins as queries, BLAST searches were performed in Phytozome to identify putative homologs in maize. Candidate sequences with an E-value ≤ 1 × 10^−5^ were retained, and redundant sequences were removed. Protein domain confirmation of the candidate members was subsequently conducted using the SMART database [31] (https://smart.embl.de/help/smart_about.shtml, accessed on 1 March 2025) and the InterPro database [32] (https://www.ebi.ac.uk/interpro, accessed on 24 March 2025). Genes encoding proteins with the characteristic GASA domain were designated as bona fide GASA family members.

To further characterize the identified ZmGASA proteins, transmembrane domains and their topological structures were predicted using the TMHMM 2.0 server (https://services.healthtech.dtu.dk/services/TMHMM-2.0, accessed on 17 May 2025). Protein length was also recorded. The theoretical isoelectric point (pI) and molecular weight (MW) of each protein were calculated using the ExPASy server, while subcellular localization was predicted with Wolf PSORT [33] (https://wolfpsort.hgc.jp, accessed on 1 June 2025).

### 4.2. Prediction of Secondary and Tertiary Structures of Maize GASA Proteins

The amino acid sequences of maize GASA family members were subjected to secondary structure prediction using the SOPMA server (https://npsa-prabi.ibcp.fr/cgi-bin/npsa_automat.pl?page=/NPSA/npsa_sopma.html, accessed on 17 June 2025). Subsequently, three-dimensional structural models of ZmGASA proteins were generated and analyzed using the SWISS-MODEL server [34] (https://www.expasy.org/resources/swiss-model, accessed on 2 July 2025).

### 4.3. Phylogenetic Analysis of GASA Family Members Across Species

A phylogenetic analysis was performed using MEGA 11 [35] software based on GASA protein sequences from maize (*Zea mays* L., 13 genes), *Arabidopsis thaliana* (11 genes), rice (*Oryza sativa*, 9 genes), sorghum (*Sorghum bicolor*, 9 genes), sugarcane (*Saccharum officinarum*, 7 genes), grapevine (*Vitis vinifera*, 10 genes), and poplar (*Populus trichocarpa*, 19 genes). The Neighbor-Joining (NJ) method was applied with 1000 bootstrap replicates to construct the phylogenetic tree.

### 4.4. Phylogenetic Tree Construction, Conserved Motif, Domain, and Gene Structure Analysis of Maize GASA Family Members

The phylogenetic relationships, conserved motifs, domains, and gene structures of ZmGASA family members were analyzed using MEGA 11, the MEME suite [36] (https://meme-suite.org/, accessed on 17 April 2025), InterPro [32], and TBtools 2.136 [37]. A phylogenetic tree was constructed in MEGA 11 based on the amino acid sequences of the 13 ZmGASA proteins using the Neighbor-Joining (NJ) method with 1000 bootstrap replicates. Conserved motifs of the 13 ZmGASA proteins were identified using MEME, while gene structure analysis was performed in TBtools [37]. Conserved domains of ZmGASA proteins were further annotated using the InterPro database. Finally, all datasets were integrated and visualized using TBtools [37] to generate a combined representation of the phylogenetic tree, gene structure, conserved motifs, and domains.

### 4.5. Cis-Acting Element Analysis of Maize GASA Gene Family Members

The promoter regions (2000 bp upstream of the transcription start site) of the 13 *ZmGASA* genes were analyzed using the PlantCARE database [38] (https://bioinformatics.psb.ugent.be/webtools/plantcare/html/, accessed on 17 April 2025) to identify putative cis-acting regulatory elements. Functional categorization and statistical analysis of the identified elements were subsequently performed, and the results were visualized using TBtools [37].

### 4.6. Synteny Analysis of Maize GASA Gene Family Across Species

Rice (*O. sativa*), sorghum (*S. bicolor*), sugarcane (*S. officinarum*), grapevine (*V. vinifera*), and poplar (*P. trichocarpa*) were selected for comparative synteny analysis with maize GASA genes. Following BLAST-based sequence alignment, syntenic gene pairs were identified, and the resulting data were visualized using TBtools [37].

### 4.7. Expression Analysis of Maize GASA Genes Under Stress Conditions

Using three maize inbred lines—B73, 082, and 107—as experimental materials (seeds provided by the Maize Research Institute of Southwest University, China), germination was conducted in a growth chamber at 25 °C via filter paper moistened with demineralized water. When seedlings reached the three-leaf stage, uniformly growing plants were selected, their roots gently rinsed, and then transferred to Hoagland nutrient solution (Coolaber Technology Co., Ltd., Beijing, China). In the preliminary experiments, three-leaf stage seedlings of the inbred line 107 were subjected to treatment with low-phosphorus. Combing with previous studies [39], treatment condition was set as follow: low-phosphorus (5 µM KH_2_PO_4_) treatment, while the normal phosphorus was 250 µM. The growth chamber was maintained at a photoperiod of 14 h light/10 h dark, day/night temperatures of 28 °C/24 °C, and 65% relative humidity. After 14 days of treatment, seedlings with consistent phenotypes and uniform growth were selected. For each treatment, four healthy plants were pooled as one biological replicate, with three biological replicates per condition.

Samples were immediately frozen in liquid nitrogen and sent to Biomarker Technologies Co., Ltd. (Beijing, China) for RNA sequencing (RNA-seq) analysis. The expression profiles of GASA family genes were analyzed using the FPKM (Fragments Per Kilobase of transcript per Million mapped reads) values provided by the sequencing company. The raw data were log_2_ (FPKM + 1)] for data normalization and subsequently visualized as a heatmap via the heatmap function implemented in TBtools [37].

For quantitative real-time PCR (qRT-PCR) analysis, cDNA was synthesized using the Plus All-in-one 1st Strand cDNA Synthesis SuperMix (gDNA Purge, E047-01B; Novoprotein, Shanghai, China), and qRT-PCR was conducted with SYBR qPCR SuperMix Plus. In each reaction, 20 µL PCR reaction system), 10 µL SYBR qPCR SuperMix Plus, 0.5 µL of each forward and reverse primer, 1 µL cDNA, and 8 µL ultra-pure water were used. The initial denaturing conditions was 95 °C for 30 s, followed by 40 cycles at 95 °C for 5 s, and 60 °C for 30 s. A melting curve was run after the PCR cycles, and the time was 95 °C for 15 s, 60 °C for 30 s, and 95 °C for 15 s. *ZmActin3* was used as the internal reference gene. Each sample was analyzed in three technical replicates, and relative expression levels were calculated using the 2*^−ΔΔCt^* method [40].

To validate these transcriptome results, primer pairs were designed for *ZmGASA06*, *ZmGASA08*, *ZmGASA09*, and *ZmGASA10* using SnapGene. However, primers designed in the gene-specific region of *ZmGASA06* or *ZmGASA09* do not amplify fragments, and primers designed in other regions cannot distinguish between homologous genes. Consequently, only ZmGASA08 and ZmGASA10 had the chance for qRT-PCR validation.

The primer sequences used were as follows: *ZmActin3*, forward 5′-TCACCCTGTGCTGCTGACCG-3′ (Tm 60 °C, 20 bp), reverse 5′-GAACCGTGTGGCTCACACCA-3′ (Tm 60 °C, 20 bp); ZmGASA08, forward 5′-TCACCACCAGCAACACCAAG-3′ (Tm 60 °C, 20 bp), reverse 5′-GCAGATGTTCTTGCGCGAGT-3′ (Tm 60 °C, 20 bp); ZmGASA10, forward 5′-ATGGCTGGATCAGGGTTCTG-3′ (Tm 60 °C, 20 bp), reverse 5′-TGGTCATGTCGCGGTAGCAT-3′ (Tm 60 °C, 20 bp).

### 4.8. Statistical Analysis

QRT-PCR data were analyzed using one-way analysis of variance (ANOVA). When significant differences were detected (*p* < 0.05), pairwise comparisons were conducted using the least significant difference (LSD) test, and the Waller–Duncan post hoc test was additionally applied to validate the robustness of multiple comparisons. All statistical analyses were performed using SPSS version 27.0, with a significance threshold set at *p* < 0.05. In the bar charts, different lowercase letters (e.g., a, b, c) indicate statistically significant differences among groups (*p* < 0.05), whereas identical letters denote no significant difference (*p* > 0.05).

## 5. Conclusions

In this study, we identified a total of 13 GASA family members in maize and performed a comprehensive genome-wide analysis, including phylogenetic relationship reconstruction, functional categorization, gene structure annotation, and conserved motif characterization. Multiple cis-acting elements associated with hormone responsiveness, stress tolerance, and light signaling suggest that GASA family members are potential involved in regulating plant growth and development. Furthermore, RNA-seq combined with qRT-PCR validation demonstrated that ZmGASA08 and ZmGASA10 were significantly upregulated under low-phosphorus treatment, implicating these two genes in nutrient-responsive pathways—particularly in phosphorus uptake, utilization, and signaling transduction. Overall, our findings lay a solid foundation for future functional characterization of ZmGASA genes and provide valuable insights into their roles in maize development and responses to nutritious cues.

## Figures and Tables

**Table 1:** Genome-wide identification and basic characteristics of ZmGASA genes.

Gene	Gene ID	Chromosome Location	CDS Length	PI	Molecular Weight (Da)	Subcellular Localization	Amino Acid Length (aa)	Transmembrane Domain	Topology of the Transmembrane Domain
*ZmGASA01*	*Zm00001eb026020_T001*	Chr1	318	6.2	11,392.27	Extracellular	106	1	i7-29o
*ZmGASA02*	*Zm00001eb050150_T001*	Chr1	288	8.84	10,232.95	Chloroplast	96	0	Na
*ZmGASA03*	*Zm00001eb050160_T001*	Chr1	282	8.75	9744.67	Chloroplast	94	1	o10-32i
*ZmGASA04*	*Zm00001eb050170_T001*	Chr1	282	8.88	9733.7	Chloroplast	94	1	o10-32i
*ZmGASA05*	*Zm00001eb050180_T001*	Chr1	432	8.18	15,327.2	Extracellular	144	1	o61-83i
*ZmGASA06*	*Zm00001eb051810_T001*	Chr1	303	9.07	10,594.2	Chloroplast	101	1	o10-27i
*ZmGASA07*	*Zm00001eb066440_T001*	Chr2	396	7.96	13,380.36	Chloroplast	132	1	o10-32i
*ZmGASA08*	*Zm00001eb080410_T001*	Chr2	348	9.12	11,904.94	Extracellular	116	1	o10-32i
*ZmGASA09*	*Zm00001eb228920_T001*	Chr5	600	8.93	22,314.63	Chloroplast	200	1	i83-102o
*ZmGASA10*	*Zm00001eb214350_T001*	Chr5	291	8.91	10,156.82	Extracellular	97	1	i7-29o
*ZmGASA11*	*Zm00001eb348690_T001*	Chr8	285	8.49	9921.59	Extracellular	95	0	Na
*ZmGASA12*	*Zm00001eb405550_T001*	Chr10	390	9.14	13,555.48	Extracellular	130	1	i7-29o
*ZmGASA13*	*Zm00001eb438860_T001*	Sca200	390	9.14	13,555.48	Extracellular	130	1	i7-29o

**Table 2:** Prediction of the secondary structure of ZmGASA proteins.

Gene	Number of α-Helix	Proportion of α-Helix	Number of Extended Strand	Proportion of Extended Strand	Number of β-Turn	Proportion of β-Turn	Number of Random Coil	Proportion of Random Coil
*ZmGASA01*	19	18.10%	14	13.33%	3	2.86%	69	65.71%
*ZmGASA02*	33	34.74%	10	10.53%	8	8.42%	44	46.32%
*ZmGASA03*	41	44.09%	4	4.30%	6	6.45%	42	45.16%
*ZmGASA04*	42	45.16%	4	4.30%	8	8.60%	39	41.94%
*ZmGASA05*	62	43.36%	11	7.69%	6	4.20%	64	44.76%
*ZmGASA06*	45	45.00%	2	2.00%	7	7.00%	46	46.00%
*ZmGASA07*	50	38.17%	8	6.11%	12	9.16%	61	46.56%
*ZmGASA08*	25	21.74%	16	13.91%	9	7.83%	65	56.52%
*ZmGASA09*	60	30.15%	32	16.08%	11	5.53%	96	48.24%
*ZmGASA10*	22	22.92%	14	14.58%	8	8.33%	52	54.17%
*ZmGASA11*	27	28.72%	9	9.57%	4	4.26%	54	57.45%
*ZmGASA12*	33	25.58%	10	7.75%	12	9.30%	74	57.36%
*ZmGASA13*	33	25.58%	10	7.75%	12	9.30%	74	57.36%

## Data Availability

The transcriptomes data of low-phosphorus treatment have been deposited in the Genome Sequence Archive of the BIG Submission Portal, National Genomics Data Center (NGDC, https://ngdc.cncb.ac.cn/) under the Bioproject (PRJCA044786).
